# Effects of biomedical messages and expert-recommended messages on reducing mental health-related stigma: a randomised controlled trial

**DOI:** 10.1017/S2045796019000714

**Published:** 2019-11-22

**Authors:** Yasutaka Ojio, Sosei Yamaguchi, Kazusa Ohta, Shuntaro Ando, Shinsuke Koike

**Affiliations:** 1 Center for Evolutionary Cognitive Sciences, Graduate School of Arts and Sciences, The University of Tokyo, 3-8-1 Komaba, Meguro-ku, Tokyo 153-8902, Japan; 2 Department of Community Mental Health & Law, National Institute of Mental Health, National Center of Neurology and Psychiatry, Kodaira, Tokyo 187-8553, Japan; 3 Department of Neuropsychiatry, The University of Tokyo, 7-3-1 Hongo, Bunkyo-ku, Tokyo 113-8655, Japan; 4 Institute for Diversity & Adaptation of Human Mind (UTIDAHM), The University of Tokyo 3-8-1 Komaba, Meguro-ku, Tokyo 153-8902, Japan; 5 UTokyo Center for Integrative Science of Human Behavior (CiSHuB), 3-8-1 Komaba, Meguro-ku, Tokyo 153-8902, Japan

**Keywords:** Common mental disorders, education psychiatric, mental health, mental illness stigma, randomised controlled trials

## Abstract

**Aims:**

Mental health-related stigma is a major challenge associated with the huge mental health treatment gap. It has remained unclear what kind of educational content is effective in reducing the stigma. Whether biomedical messages (BMM) about mental illness are effective or harmful in decreasing stigma is controversial. To investigate whether BMM can improve practically useful knowledge of mental illness, comparably to recommended messages (RCM) advocated by experts, of types such as ‘recovery-oriented’, ‘social inclusion/human rights’ and ‘high prevalence of mental illnesses’ through a randomised controlled trial (RCT).

**Method:**

This study is an individual-level RCT with a parallel-group design over 1 year, conducted in Tokyo, Japan. A total of 179 participants (males *n* = 80, mean age = 21.9 years and s.d. = 7.8) were recruited in high schools and universities, and through a commercial internet advertisement in June and July 2017, without any indication that the study appertained to mental health. Participants were allocated to the BMM and RCM groups. They underwent a 10-min intervention, and completed self-report questionnaires during baseline, post-test, 1-month follow-up and 1-year follow-up surveys. The primary outcome measures were practically useful knowledge of mental illness at the post-test survey using the Mental Illness and Disorder Understanding Scale (MIDUS). Analysis was conducted in October 2018.

**Results:**

Both groups demonstrated improved MIDUS score in the post-test survey, and showed similar intervention effects (*F*_(1, 177)_ = 160.5, *p* < 0.001, *η*^2^ = 0.48). The effect of the interventions continued until the 1-year follow-up survey (*B* [95% CI] = −2.56 [−4.27, −0.85], *p* < 0.01), and showed no difference between groups. The reported adverse effect that BMM increase stigma was not confirmed.

**Conclusions:**

BMM may have a positive impact on stigma, comparable to RCM. These findings may encourage reconsideration of the content of messages about mental health, as it is indicated that combining BMM and RCM might contribute to an effective anti-stigma programme.

## Introduction

Overcoming mental health related-stigma is an international issue. Mental health-related stigma has a major negative impact on help-seeking behaviour, and is a barrier to treatment and recovery for people with mental health problems (Ando *et al*., 2013; Gronholm *et al*., 2017; Schnyder *et al*., 2017). More than 70% of persons who need mental health service have lacked access to care (Kohn *et al*., 2004), despite mental illnesses being treatable (Insel, 2009). The mental health treatment gap increases the enormous burden of mental illnesses worldwide (Whiteford *et al*., 2013). Decreasing mental health-related stigma can lead to diminishing the mental health treatment gap (Kohn *et al*., 2004).

Over the past three decades, many studies have examined the effects of anti-stigma interventions. Mental health-related stigma is constructed through multiple aspects such as knowledge, attitudes and behaviour (Corrigan and Watson 2002; Rüsch *et al*., 2005; Thornicroft *et al*., 2007). A lack of knowledge of mental illness is associated with negative attitudes toward mental illnesses, which contribute to actual discriminatory behaviour (Corrigan *et al*., 2003). Since more emphasis is placed on positive views of treatment and recovery process in the recent clinical strategy for mental illness (Bertolote and McGorry, 2005), knowledge of mental illness can also be divided into positive and evidence-based knowledge aspect (practically useful knowledge of mental illness) and negative stereotyped aspect (e.g., ‘people with mental illness are dangerous’) (Corrigan and Shapiro, 2010; Koike *et al*., 2015; Koike *et al*., 2018*a*, 2018*b*). The practically useful knowledge has been considered to encourage better understanding toward people with mental health problems and may help-seeking behaviour (Rüsch *et al*., 2011). The educational approach has been recognised as an effective intervention for reducing mental health-related stigma, mainly through knowledge (or stereotype) (Yamaguchi *et al*., 2011; Thornicroft *et al*., 2016). Randomised controlled trials (RCTs) have shown that social contact or contact-based intervention (e.g., filmed social contact) is the most effective strategy for decreasing stigma, especially with regard to mental health-related attitudes and/or behavioural intentions (Thornicroft *et al*., 2016; Koike *et al*., 2018*a*, 2018*b*; Yamaguchi *et al*., 2019). Combining social contact with knowledge-based education, rather than social contact alone, appears to enhance the effect of decreasing stigma (Griffiths *et al*., 2014). However, it is still debatable what kind of educational content is effective in reducing stigma.

A consensus development study showed an expert opinion-based recommendation for designing educational interventions to tackle mental health-related stigma (Clement *et al*., 2010). Expert opinions have recommended the following types of messages: ‘See the person’ (equal to social contact), ‘recovery-oriented’, ‘social inclusion/human rights’ and ‘high prevalence of mental illnesses’ (Clement *et al*., 2010). Mental health literacy intervention, mainly focusing on promoting help-seeking efficiency, has also been recognised as an effective approach for decreasing stigma (Kutcher *et al*., 2016a, 2016b; Wei *et al*., 2013). The educational contents in such programmes were diverse and mainly consisted of recommended messages (RCM) and some information about the biological basis for psychiatric disorders including, for example, that ‘the basic functions of the brain as the organ that controls thinking, moods and behaviour’ and ‘mental disorders are the result of changes that arise in usual brain function as a result of a complex interplay between a person's genes and environment’ (Watson *et al*., 2004; McLuckie *et al*., 2014; Milin *et al*., 2016). Past trial-based studies have produced evidence that biomedical information may contribute to decreasing stigma through affecting the stereotype by improving scientific-based medical knowledge, for example about the treatability of mental illnesses (Lebowitz and Ahn, 2012; Han and Chen, 2014). Biological elements of mental illness enhance cognitive interpretations and/or judgments to a medicalised framing, and are therefore considered an effective anti-stigma strategy in healthcare (Knaak *et al*., 2015; Ungar *et al*., 2016). Expert opinions and observational research findings have, however, suggested that biological explanations (e.g., about biological causes of mental illnesses, including genetics or chemical imbalance) may increase stigma and in part have been harmful during the past decade (Clement *et al*., 2010; Angermeyer *et al*., 2011; Schomerus *et al*., 2012). Whether biomedical information about mental illness itself is effective or harmful in decreasing stigma is controversial.

To the best of our knowledge, there have been no RCT testing whether biomedical messages (BMM) about mental illness are more effective in reducing stigma compared with the RCM. Our preliminary pilot study showed that BMM improved practically useful knowledge of mental illness more than RCM. In the present study, we aim to investigate whether a 10-min film with BMM improves more practically useful knowledge of mental illness towards people with mental health problems measured using the Mental Illness and Disorder Understanding Scale (MIDUS) from the baseline to the post-test survey compared with the RCM. As a secondary outcome, we conducted follow-up surveys for 1 year to explore the maintenance of the intervention effects.

## Methods

### Study design

We conducted an individual-level RCT with a parallel-group design. The present study included baseline, post-test, 1-month follow-up and 1-year follow-up surveys. We set three days in June and July 2017 for the baseline survey. Participants were individually allocated to either the BMM video lecture group or the RCM video lecture group, before the baseline survey. Each participant completed the baseline survey (mean duration 20 min), individually viewed one of the 10-min intervention videos on a personal computer, and completed the post-test survey immediately after the intervention. The intervention and the baseline and post-test surveys were completed in a room at The University of Tokyo. The 1-month and 1-year follow-ups were administered by means of a web survey in order to avoid attrition. All the surveys were conducted using anonymous, self-administered questionnaires. The allocation was masked to the researchers involved in processing and analysing the data until all the participants had completed their baseline survey. The researchers received the data from each assessment and allocation of each participant after the participant had completed the post-test survey. Therefore, this RCT was categorised as assessor-blinded in registration. The present study was registered at the University Hospital Medical Information Network Clinical Trial Registration before the start of the initial survey (trial number: UMIN000027727) and underwent no further methodological changes after registration.

### Participants

We recruited a general population including high school students, undergraduate and graduate students, young adults and adults by means of recruitment boards in several high schools and universities and a commercial internet advertisement. Participants were recruited without being given any information indicating this was a mental health-related survey or trial to avoid influencing the results due to participants' interest in mental health, but it was explained that the project explored the effects of different learning methods. The study set the following exclusion criteria: those who are unable to read and write in Japanese at a level equivalent to graduation from junior high school in Japan. Ethical approval was obtained from the Research Ethics Committee of the Department of Arts and Sciences, The University of Tokyo (approval no. 507-2). All the participants provided informed written consent after receiving a full explanation of the study, including the detailed study settings and main purpose.

### Sample size

We estimated an initial sample size of 76 participants in each group, based on the results of a preliminary pilot study in which 21 participants were allocated to each of the BMM and RCM groups. The sample size calculation was conducted based on a 3.00 mean difference between the group in the pilot study. Detecting this difference with *α* = 0.05 and *β* = 0.80 would require 76 participants. To allow for a dropout rate up to 25% and a reduced effect for 1-year, the final estimation was 95 participants for each group (190 in total).

### Randomisation and blinding

Random allocation was conducted by an opaque and sealed envelope method. A research assistant (K.O.), independent from the interventions, assessments and data analysis, generated random permuted blocks with block sizes of four or six stratified by sex using a website (www.randomization.com). The research assistant produced the allocation sequence and all the envelopes before the start of the trial. The authors conducted enrolment and assignment without any information from the survey. As each participant was assigned to one of the groups during the baseline assessment, concealment of the allocation of each participant was maintained before completion of the assessment at baseline. The allocation was masked to the researchers involved in processing and analysing the data until all the participants had completed the baseline survey.

### Interventions

Following completion of the baseline survey, participants received individual laptop computers containing one of two 10-min interventions assigned according to the groups. Table 1 shows the contents of each film. The BMM group viewed a film covering ‘Biological mechanism of mental illnesses’, ‘Pharmacological mechanism’ and ‘Gene-environment interaction’. The film for the RCM group covered the following: (1) high prevalence of mental illnesses, (2) recovery-oriented messages and (3) social inclusion/human rights messages (Clement *et al*., 2010). These messages have a confirmed positive impact on reducing negative stereotype (Thornicroft *et al*., 2016; Koike *et al*., 2018*a*, 2018*b*; Yamaguchi *et al*., 2019). Filmed social contacts were not included in either BMM or RCM. The details of each film are shown in Table 1. Both films were produced by the authors, all of whom had sufficient experience in community mental health services. Draft intervention films were used in the preliminary pilot study. We received feedback on understandability and impact of all the clips in the films using an 11-Likert scale. We also asked the participants about their impressions, sympathy, understanding of the participant and the change of impression of mental health problems through the films, using an 11-Likert scale. We revised the films according to the feedback. The films used in the trial were also evaluated in a similar manner, and there was no significant difference between the films in mean understandability (BMM: 7.62 [s.d. = 1.49], RCM: 7.83 [1.31], *p* = 0.31) or impact (BMM: 6.94 [1.60], RCM: 7.09 [1.50], *p* = 0.53).

**Table 1.** Contents of the interventions in this trial

Type of messages	Title of clip	Explanation
*Common introduction for both groups*		
	Definition of mental illness and psychological symptoms	Typical and common symptoms of mental health problems or mental illness (e.g., anxiety, concern, fear, irritation, impatience, insomnia and daytime sleepiness.)
*RCM video lecture*		
High prevalence of mental illnesses	Mental illness as a common disease	Point prevalence of mental illness (e.g., One in 11 people have a mental illness.) Lifetime prevalence of mental illness (e.g., One in five people will suffer from mental illness.)
	Onset of mental illness	Peak age at onset of mental illness (e.g., 50% of mental illness onset prior to the age of 14, and 75% onset by 25.)
	The burden of mental illness in our society	Calculated in Disability-Adjusted Life Years, the burden of disease in mental illness accounts for the largest proportion in all diseases. Mental health problems account for about 70% of the health problems of adolescents.
Recovery-oriented	Early detection and treatment for recovery	Not only self-care, but also the support of friends, family members and the surrounding community is essential for recovery. Early detection and treatment are effective for recovery from mental health problems. Support includes the understanding of family and friends, and counselling and environmental considerations A gradual return to school or workplace is effective for recovery in mental illness.
Social inclusion/human rights	Rehabilitation as an effective approach for recovery	People with mental illness have the right to receive assistance in order to work and continue to be employed. The employment system for people with disabilities has been improved, and the number of people working is rapidly increasing in people with mental illness.
	Mutual support society	People with mental illness and other disabilities can live in the same community with understanding and support from people in the community.
*BMM video lecture*		
Biological mechanism of mental illnesses	Mental illness as a disease of the brain	Advances in brain imaging technology. Studies have shown that people with mental illnesses have some alterations in the brain volume and function compared to healthy control subjects. Mental illness is thought to occur due to brain malfunction similar to physical illnesses due to internal organ malfunction.
	Brain, synapse and neurotransmitters	Neurons exchange information with other neurons by means of electrical signals. The connection between nerve cells is called a synapse. Neurotransmitters are released by the electric information in a nerve cell. Neurotransmitters (adrenaline, dopamine, serotonin, GABA etc.) transmit information to the next nerve cell.
	Neurotransmitters and mental health status	One of the potential causes of mental health problems is the imbalance of neurotransmitters in the brain.
Pharmacological mechanism	Pharmacological mechanism of psychotropic agents	Psychiatric drugs potentially improve synaptic imbalance and mental health status. Mental health problems can be treated with psychiatric drugs.
Gene-environmental interaction	Genetic and environmental factors in mental illness	Genetic studies have suggested that around 50% of cause of mental illnesses are thought to be genetic factors. Most mental health problems, mental illnesses and personality problems are affected by the interaction of many genes and environmental factors. Specific factors in both genes and the environment have yet to be found.

### Outcome measures

We assessed the change in stigma-related outcomes, according to the MIDUS, the Japanese version of the Reported and Intended Behaviour Scale (RIBS-J) and help-seeking intention scales in the baseline, post-test, 1-month follow-up and 1-year follow-up surveys. For the primary outcome measure, we used the MIDUS for the post-test survey.

#### MIDUS

The MIDUS was employed to assess practically useful knowledge of mental illness. The factor analysis revealed the three subscales including treatability of illness (e.g., ‘Mental illness is treatable’), efficacy of medication (e.g., ‘Medication is effective in improving symptoms’) and social recognition of illness (e.g., ‘Mental illnesses are very common’) (Tanaka, 2003). It consists of 15 items on a five-point Likert scale (range 0 to 60, a lower score representing more practically useful). The MIDUS was originally developed in Japan, and its factorial validity and moderate internal consistency have been confirmed (Tanaka, 2003). Cronbach's *α* was 0.79 in the baseline survey in the present sample.

#### RIBS-J

The RIBS-J consists of four binary items for past experience with people with mental health problems (RIBS-J past, range 0–4; ‘Yes’ = 1, ‘No’ = 0, ‘Don't know’ = 0; higher scores represent more social contact) and four items for future behavioural intentions on a 5-point Likert scale (RIBS-J future, range 4–20; ‘Strongly agree’ = 5 to ‘Strongly disagree’ = 1; higher scores indicate a more positive intention, such as ‘In the future, I would be willing to live with someone with a mental health problem’) (Evans-Lacko *et al*., 2011). Both the original and Japanese versions have good validity and reliability (Evans-Lacko *et al*., 2011; Yamaguchi *et al*., 2014). Cronbach's *α* was 0.68 and 0.84 in the baseline survey in the present sample for the RIBS-J past and the RIBS-J future, respectively.

#### Help-seeking

The following two questions evaluated help-seeking intention and attitude to mental illness. First, whether participants had the intention to seek help from mental health professionals was measured by the following question: ‘If you felt that you had a mental health problem, how likely would you be to go to mental health professionals for help?’ Responses were rated on a Likert scale of 1 to 5, with higher scores indicating a greater likelihood of seeking help. The mean ± s.d. score for the 1751 adults was 4.2 ± 1.1 (Rüsch *et al*., 2011). Second, whether participants felt comfortable disclosing a mental illness to friends or relatives was measured by the question: ‘In general, how comfortable would you feel talking to a friend or family member about your mental health, for example, telling them you have a mental health diagnosis and how it affects you?’ Scores range from 1 to 7, with higher scores reflecting greater comfort with disclosure. The mean ± s.d. score for the 1751 adults was 5.1 ± 1.9 (Rüsch *et al*., 2011).

### Statistical analysis

For the primary outcome, we used a repeated *t*-test for the MIDUS scale change between the group. Effect size *η*^2^ was calculated, which was graded as 0.01 = small, 0.06 = medium and 0.14 = large. For secondary outcomes to test the differences in stigma scales over the four survey time points, we applied a random effect of intercept and slope in a mixed effect model with full maximum likelihood estimation. For the effect size of the mean difference between the group in each assessment point, through computing Cohen's *d* which was graded as 0.20 = small, 0.50 = medium and 0.80 = large. We also added sex and age as covariates to the models, because these variables have been regarded as potential confounders in previous stigma studies (Holzinger *et al*., 2012; Yamaguchi *et al*., 2013). For age, the participants were categorised into the following three subgroups: younger (aged 15–18 years, *n* = 58), mid-range (aged 18–21 years, *n* = 63) and older (aged 21–58 years, *n* = 58). As sensitivity analyses, we adjusted for sex, age, experience of having personal mental health problems, experience of attending a lecture on mental health and experience of watching media describing people with mental health problems, which were regarded as potential confounders or effect modifiers in past studies. For the proportion of mental health-related experiences during the follow-up periods, changes from baseline to 1-year follow-up in each group were tested using McNemar's test, and comparisons between groups at 1-year follow-up survey were tested using the χ^2^ test. To check for any an adverse effect of the interventions, we defined a 25% or more decrease in the RIBS-J future score from the baseline to each follow-up as an increase in stigma (Koike *et al*., 2018*a*, 2018*b*) and compared the number of those who demonstrated an increase in stigma between the two groups using Fisher's exact test. Statistical significance was set at 5% of variance. All analyses were carried out in October 2018. Statistical analyses were conducted using Stata/SE 14.2, StataCorp, 2017.

## Results

**Fig. 1.**
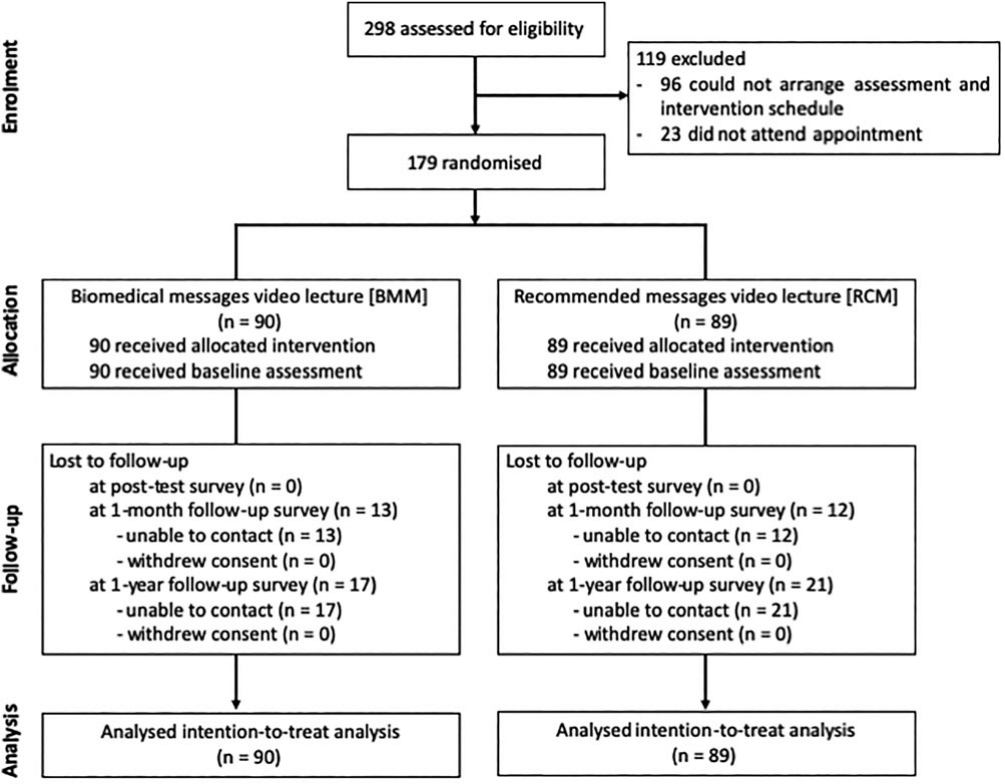
CONSORT flow diagram of the study.

There were 298 applicants for this trial. Of these, 179 persons participated in the study and were randomly assigned to the BMM (*n* = 90) and RCM groups (*n* = 89) (Fig. 1). There were no differences in demographic characteristics or scores for outcome measures at baseline between the groups (Table 2).

**Table 2.** Characteristics of participants in this trial

	BMM video lecture	RCM video lecture	Statistical values^a^	
	*n* = 90	*n* = 89		
Sex, female, *n* (%)	50 (55.56)	49 (55.06)	*χ*^2^ = 0.01	*p* = 0.95
Age, mean (s.d.)	21.81 (7.67)	21.99 (7.95)	*t* = *−*0.15	*p* = 0.88
Past experience of				
Having mental health problems, *n* (%)	21 (23.33)	24 (26.97)	*χ*^2^ = 1.26	*p* = 0.53
Receiving care/treatment from professionals, *n* (%)	11 (12.22)	14 (15.73)	*χ*^2^ = 0.46	*p* = 0.53
Attending a lecture about mental health problems, *n* (%)	38 (42.22)	31 (34.83)	*χ*^2^ = 1.03	*p* = 0.36
Viewing media describing an individual with mental health problems^b^, *n* (%)	72 (80.00)	65 (73.03)	*χ*^2^ = 1.21	*p* = 0.29
Seeking help from family members and close friends for mental health problems, *n* (%)	24 (26.67)	21 (23.60)	*χ*^2^ = 0.22	*p* = 0.73
MIDUS, mean (s.d.)	16.60 (6.93)	17.84 (7.53)	*t* = *–*1.15	*p* = 0.25
RIBS-J				
Reported behaviour (past) subscale, mean (s.d.)	0.62 (0.89)	0.79 (1.06)	*t* = *–*1.12	*p* = 0.26
Intended behaviour (future) subscale, mean (s.d.)	12.49 (3.73)	12.04 (3.61)	*t* = 0.81	*p* = *0*.42
Intention to seek help from mental health professionals, mean (s.d.)	3.76 (0.93)	3.73 (0.89)	*t* = 0.19	*p* = 0.85
Intention to disclose, mean (s.d.)	3.12 (1.55)	2.97 (1.59)	*t* = 0.66	*p* = 0.51

MIDUS, The Mental Illness and Disorder Understanding Scale; RIBS-J, The Japanese version of the Reported and Intended Behaviour Scale.

a Group differences were tested using the *t*-test for continuous variables and using the *χ*^2^ test for categorical variables.

b Media include television, newspapers, internet, etc.

### Effects of BMM on reducing the practically useful knowledge of mental illness-related stigma

**Fig. 2.**
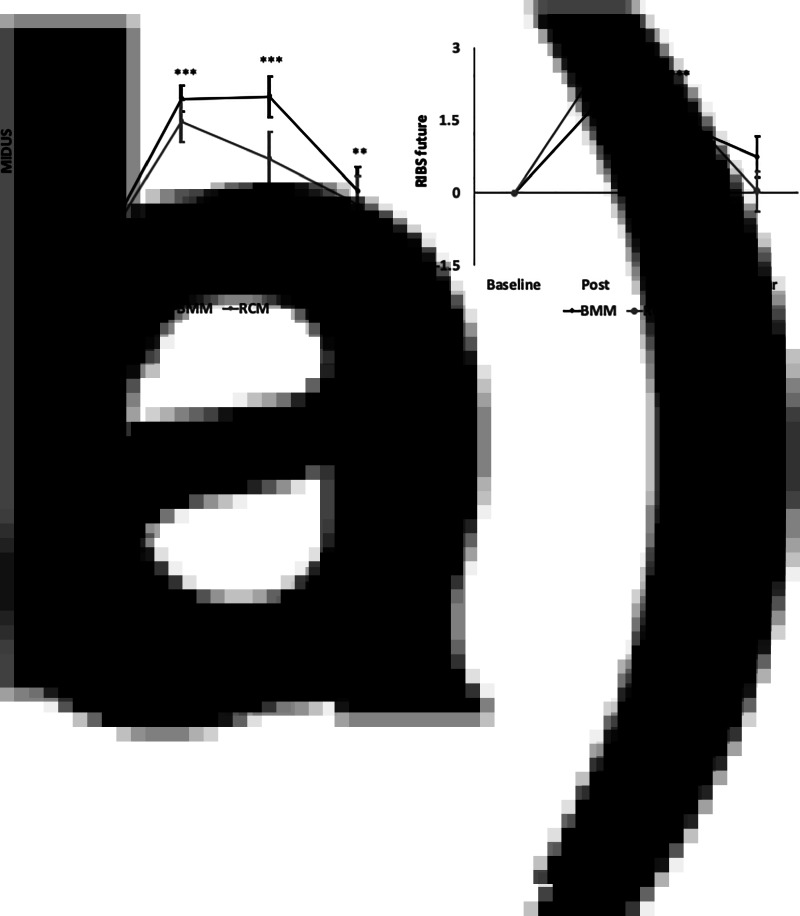
Change in the MIDUS (*a*) and RIBS-J future (*b*). Bars show standard error. Significant differences and interactions are shown (**p < 0.01; ***p < 0.001). (*a*) The main effect of time for the MIDUS was significant in both BMM (black line) and RCM (grey line) in post-test, 1-month follow-up and 1-year follow-up surveys compared with the baseline. (*b*) The main effect of time for the RIBS was significant in both BMM (black line) and RCM (grey line) in post-test and 1-month follow-up surveys compared with baseline.

As the primary outcome, repeated measure analysis revealed that the main effect of time from baseline to post-test survey was significant for the MIDUS (*F*_(1, 177)_ = 160.5, *p* < 0.001, *η*^2^= 0.48), but not the main effect of group (*F*_(1, 177)_ = 3.12, *p* = 0.08, *η*^2^ = 0.02) or the group × time interaction (*F*_(1, 177)_ = 0.88, *p* = 0.35, *η*^2^ = 0.005). As secondary outcomes for the MIDUS at the 1-month follow-up and the 1-year follow-up, the main effect of time was significant with better scores from baseline to the 1-month follow-up (*B* [95% CI] = −5.87 [−7.44, −4.30], *p* < 0.001) and to the 1-year follow-up (*B* [95% CI] = −2.56 [−4.27, −0.85], *p* < 0.01) (Fig. 2 and online Supplementary Table S1). In both groups, the highest effect sizes were observed in the post-test survey. There was no significant main effect of group (*B* [95% CI] = 1.24 [ − 0.83, 3.31], *p* = 0.23) or group × time interaction in the post-test (*B* [95% CI] = 0.84 [−1.01, 2.69], *p* = 0.37), the 1-month follow-up (*B* [95% CI] = 2.20 [−0.02, 0.42], *p* = 0.05) or 1-year follow-up survey (*B* [95% CI] = 0.67 [−1.78, 3.13], *p* = 0.59). The analysis with MIDUS subscales showed that the group × time interaction for the ‘efficacy of medication’ subscale was significant in the post-test survey (B [95% CI] = 2.76 [1.71, 3.81], *p* < 0.001), the 1-month follow-up (B [95% CI] = 1.89 [0.75, 3.03], *p* < 0.01) and the 1-year follow-up survey (B [95% CI] = 1.39 [0.03, 2.75], *p* < 0.05), indicating that the BMM group underwent a greater change than the RCM group (online Supplementary Table S2).

### Effects of BMM on reducing the behavioural intention of mental illness-related stigma

For the RIBS-J future subscale, the main effect of time was significant with better scores in the post-test survey (*B* [95% CI] = 1.81 [1.22, 2.40], *p* < 0.001), in the 1-month follow-up (*B* [95% CI] = 1.51 [0.90, 2.12], *p* < 0.001), but not in the 1-year follow-up survey (*B* [95% CI] = 0.69 [−0.06, 1.43], *p* = 0.07). In both groups, the highest effect sizes were also observed in the post-test survey. There was no significant main effect of group (*B* [95% CI] = −0.44 [−1.44, 0.55], *p* = 0.38) or group × time interaction in the post-test (*B* [95% CI] = −0.72 [−0.11, 1.55], *p* = 0.09), the 1-month follow-up (*B* [95% CI] = 0.24 [−0.62, 1.11], *p* = 0.58) or the 1-year follow-up survey (*B* [95% CI] = −0.72 [−1.80, 0.35], *p* = 0.19).

### Effects of BMM on the mental illness-related experience

For the RIBS-J past subscale, the main effects of time and group were not significant. The group × time interaction was significant (*B* [95% CI] = −0.38 [−0.66, −0.10], *p* < 0.01). The change in score from baseline to 1-year follow-up survey was greater in the BMM group compared with the RCM (online Supplementary Table S1). There were no significant changes in the proportions of participants who had other mental health-related experiences, except for ‘attending a lecture about mental health problems’ in RCM, during the 1-year follow-up in both groups, and no difference in the proportions at the 1-year follow-up survey between the groups (online Supplementary Table S3).

### Effects of BMM on help-seeking intention

For help-seeking intention, including ‘intention to seek help’ and ‘intention to disclose’, significant associations were seen in the main effects of time in the post-test survey in intention to seek-help (*B* [95% CI] = 0.22 [0.07, 0.37], *p* < 0.01), and intention to disclose in the 1-month follow-up survey (*B* [95% CI] = 0.33 [0.04, 0.63], *p* < 0.05). The group × time interaction was significant in intention to disclose in the post-test survey (*B* [95% CI] = 0.32 [0.02, 0.62], *p* < 0.05) (online Supplementary Table S4), indicating that the RCM group underwent a greater change than the BMM group.

### Effect of demographic characteristics on the difference in stigma between the groups

A significant interaction effect of group × time × sex for RIBS-J future, indicating that the female participants in the RCM group had a greater change compared with the males from the baseline to 1-month follow-up (*B* [95% CI] = 1.83 [0.11, 3.54], *p* < 0.05) and 1-year follow-up surveys (*B* [95% CI] = 2.27 [0.12, 4.42], *p* < 0.05) (online Supplementary Fig. S1). Among the three age groups, the sustained effect of each intervention on intention to disclose, and on help-seeking intention, was also significantly different, indicating that the younger participants in the RCM group had a greater change compared with the older participants from the baseline to post-test (*B* [95% CI] = −0.93 [1.66, −0.19], *p* < 0.05) and 1-year follow-up surveys (*B* [95% CI] = −1.72 [−3.07, −0.37], *p* < 0.05) (online Supplementary Fig. S2). Sensitivity analyses conducted after adjusting for demographic variables confirmed similar results in the primary and secondary outcomes.

### Difference in the adverse effect

The differences in the prevalence of an adverse effect were not significant at any of the three times of the surveys (Table 3).

**Table 3.** Prevalence of adverse effect

	BMM video lecture	RCM video lecture	*p-*values^a,^^b^
	*n* (%)	*n* (%)	
Post-test	0 (0)	5 (5.6)	*p* = 0.029
1-month follow-up	1 (1.3)	3 (3.9)	*p* = 0.620
1-year follow-up	10 (13.7)	8 (11.8)	*p* = 0.804

The adverse effect was defined using a 25% or more decrease (worsening) of the RIBS-J future score from the baseline in each survey.

a Group differences were tested using Fisher's exact test.

b The statistical significance threshold was adjusted to 0.05/3 = 0.017 with Bonferroni correction.

## Discussion

The present trial shows that, contrary to the hypothesis, the 10-min BMM had a similar effect on improving practically useful knowledge of mental illness to the RCM. The results are inconsistent with the previous evidence (Angermeyer *et al*., 2011; Schomerus *et al*., 2012), as both of the types of messages show similar effects on improving a wide range of stigma, including practically useful knowledge, behavioural intention and help-seeking intention. The reported adverse effect that BMM increase stigma was not confirmed. Thus, we demonstrate that BMM about mental health problems also work effectively in improving stereotype and behavioural intention, to the same extent as RCM.

Contrary to previous evidence, BMM had a positive impact on improving mental health-related stigma, as did RCM, and had little adverse effect. A meta-analysis shows the negative association between BMM and public acceptance in schizophrenia (Angermeyer *et al*., 2011), but not in depression (Schomerus *et al*., 2012). In the current study, typical and common symptoms of mental health problems or mental illness were explained as the definition of mental illness and psychological symptoms without disease-specific explanation. ‘Efficacy of medication’ may also be essential information for decreasing mental health-related stigma, although it is a simple message (Lebowitz and Appelbaum, 2018). As a message of ‘Efficacy of medication’, the BMM included ‘Mental health problems can be treated with psychiatric drugs’ (Table 1). Most of the negative effects of biomedical explanations in the previous studies appear to engender the perception that mental illnesses are deterministically and immutably caused by biological abnormalities (Lebowitz and Appelbaum, 2018). Based on the theoretical consequence of generating the stigma (Corrigan and Watson 2002; Rüsch *et al*., 2005; Thornicroft *et al*., 2007), negative stereotype may reduce beliefs about blameworthiness but may increase concerns about dangerousness or unpredictability of illness that may then influence behaviours. Although we did not include items about such negative belief, the results showed these detrimental effects can be ameliorated by informing people about the existence of effective treatments for mental illness including the efficacy of medication as biological factors involved in psychopathology. The current findings suggest that combining biomedical explanations with information about effective treatments for mental illness appears to decrease the mental health-related stigma.

The results, however, do not support our hypothesis despite collecting the number of samples calculated from the preliminary pilot study, because the mean difference of the MIDUS score in BMM was smaller than that assumed from the preliminary pilot study. It might be argued that the failure to detect significant aggregate effects in the analyses reflects low statistical power. The calculated effect size might have been overestimated because the number of participants in the preliminary pilot study was relatively small (*n* = 21).

We found that the maintenance effects of intervention might vary according to sex or age in participants who received RCM. The female participants and younger participants (aged 15–18 years) who received the RCM had more improvement in the behavioural intention and help-seeking intention, respectively. In the content of the RCM, ‘recovery-oriented’ and ‘social inclusion/human rights’ messages may promote empathy (Table 1). These facts, on average, may correspond with the tendency for learning styles with individual characteristics, such as females being empathetic (Christov-Moore *et al*., 2014) and adolescents being highly emotionally sensitive (Crone and Konijn, 2018). For example, the use of drama, animation and peer story-telling as an anti-stigma method to stimulate empathy and emotion may be effective in females and adolescents. Empirical data about responses to anti-stigma education that are unique to individual characteristics (e.g., sex or age) are limited, therefore the accumulation of research findings is necessary.

The improvement in both groups following the 10-min video education peaked immediately after the intervention, and statistically maintained its effect for 1-month in behavioural intentions and 1-year in knowledge regardless of message types. There are two considerations regarding these effects. First, these effects may include the effects of other mental health education and information. But in the present data, no difference was found in the proportions of participants who had mental health-related experiences during the 1-year follow-up between the groups. As a positive impact of the brief intervention on participants, it might have increased the exposure to other mental health education and information after the intervention. Second, the effect size gradually declined over the follow-up period. The previous RCT suggested that repeated education may be essential to maintain or boost the effects (Yamaguchi *et al*., 2019). Repeated classroom-based educational intervention may help to enhance and sustain effects through the influence of friends or colleagues (McLuckie *et al*., 2014). In the present study, we found that BMM is also available for the purpose of educational series and may provide a range of lecture contents. In future study, it may be important to discover how to implement long-term interventions that utilise the content of both BMM and RCM.

### Strengths of this research

We recognise that evaluating information in order to identify the key elements for an anti-stigma programme using RCT as a scientific methodology is the greatest strength of this research. Development and implementation of educational programmes are often based on expert opinion, without the process of gathering scientific evidence. Researchers should examine the content and effectiveness of the programme according to strict, scientific rules to make an appeal to policymakers and authorities.

### Limitations of this research

We also recognised several limitations that should be taken into consideration. First, because all the measurements were carried out using self-reported questionnaires, a possible methodological limitation, including social desirability bias, may have influenced the present findings. Mental health-related information from lectures and the media, and the personal experiences of participants and people close to them during the follow-up period may have improved the scores. However, the confidentiality and anonymity of the self-report questionnaires were confirmed, and the effects of the interventions were compared with controls. Therefore, these biases may have had less influence on the findings. Second, we used two single items for investigating the intention of help-seeking, which were used in a previous study and not evaluated for reliability and validity with a large number of subjects. Third, although no information relating to mental health problems was provided to the participants on recruitment, the intervention was conducted in Tokyo, Japan during the daytime. In addition, all the participants were Japanese. Social and cultural differences may have influenced the effect of the intervention (Angermeyer *et al*., 2011). Therefore, the current findings cannot easily be generalised to the wider population, and different results might be obtained from different demographic groups.

## Conclusions

Our RCT shows that BMM have a positive impact on mental health-related stigma, comparable to that of RCM. The results provide helpful indications for the development of an anti-stigma educational programme. Previous anti-stigma programmes avoided including BMM and depended on expert opinions and other recommendations. The current findings may encourage reconsideration of this. Future anti-stigma programmes or campaigns with well-circumscribed and mixed biomedical and RCM could diminish the ‘mental health treatment gap’.

## Supplementary material

For supplementary material accompanying this paper visit https://doi.org/10.1017/S2045796019000714. click here to view supplementary material

## Data

Not all the data are freely accessible because no informed consent was given by the participants for open data sharing, but we can provide the data used in this study to researchers who want to use them, following approval by the ethics committee of the University of Tokyo.

## References

[ref1] Ando S, Yamaguchi S, Aoki Y and Thornicroft G (2013) Review of mental-health-related stigma in Japan. Psychiatry and Clinical Neurosciences 67, 471–482. 24118217 10.1111/pcn.12086

[ref2] Angermeyer MC, Holzinger A, Carta MG and Schomerus G (2011) Biogenetic explanations and public acceptance of mental illness: systematic review of population studies. British Journal of Psychiatry 199, 367–372. 10.1192/bjp.bp.110.085563 22045945

[ref3] Bertolote J and McGorry P (2005) Early intervention and recovery for young people with early psychosis: consensus statement. British Journal of Psychiatry. 48, s116–s119. 10.1192/bjp.187.48.s116 16055800

[ref4] Christov-Moore L, Simpson EA, Coudé G, Grigaityte K, Iacoboni M and Ferrari PF (2014) Empathy: gender effects in brain and behavior. Neuroscience and Biobehavioral Reviews 4, 604–627. 10.1016/j.neubiorev.2014.09.001 PMC5110041 25236781

[ref5] Clement S, Jarrett M, Henderson C and Thornicroft G (2010) Messages to use in population-level campaigns to reduce mental health-related stigma: consensus development study. Epidemiology Psychiatric Sciences 19, 72–79. 10.1017/s1121189x00001627 20486426

[ref6] Corrigan PW and Shapiro JR (2010) Measuring the impact of programs that challenge the public stigma of mental illness. Clinical Psychology Review 30, 907–922. 20674114 10.1016/j.cpr.2010.06.004 PMC2952670

[ref7] Corrigan PW and Watson AC (2002) Understanding the impact of stigma on people with mental illness. World Psychiatry 1, 16–20. 16946807 PMC1489832

[ref8] Corrigan PW, Markowitz FE, Watson A, Rowan D and Kubiak MA (2003) An attribution model of public discrimination towards persons with mental illness. Journal of Health and Social Behavior 44, 162–179. 12866388

[ref9] Crone EA and Konijn EA (2018) Media use and brain development during adolescence. Nature Communications 9, 588. 10.1038/s41467-018-03126-x PMC5821838 29467362

[ref10] Evans-Lacko S, Rose D, Little K, Flach C, Rhydderch D, Henderson C and Thornicroft G (2011) Development and psychometric properties of the reported and intended behaviour scale (RIBS): a stigma-related behaviour measure. Epidemiology Psychiatric Sciences 20, 263–271. 21922969 10.1017/s2045796011000308

[ref11] Griffiths KM, Carron-Arthur B, Parsons A and Reid R (2014) Effectiveness of programmes for reducing the stigma associated with mental disorders. A meta-analysis of randomized controlled trials. World Psychiatry 13, 161–175. 24890069 10.1002/wps.20129 PMC4102289

[ref12] Gronholm PC, Thornicroft G, Laurens KR and Evans-Lacko S (2017) Mental health-related stigma and pathways to care for people at risk of psychotic disorders or experiencing first-episode psychosis: a systematic review. Psychological Medicine 47, 1867–1879. 28196549 10.1017/S0033291717000344

[ref13] Han DY and Chen SH (2014) Reducing the stigma of depression through neurobiology-based psychoeducation: a randomized controlled trial. Psychiatry and Clinical Neurosciences 68, 666–673. 24521323 10.1111/pcn.12174

[ref14] Holzinger A, Floris F, Schomerus G, Carta MG and Angermeyer MC (2012) Gender differences in public beliefs and attitudes about mental disorder in western countries: a systematic review of population studies. Epidemiology Psychiatric Sciences 21, 73–85. 22670415 10.1017/s2045796011000552

[ref15] Insel TR (2009) Disruptive insights in psychiatry: transforming a clinical discipline. Journal of Clinical Investigation 119, 700–705. 10.1172/JCI38832 PMC2662575 19339761

[ref16] Knaak S, Ungar T and Patten S (2015) Seeing is believing: biological information may reduce mental health stigma amongst physicians. Australian & New Zealand Journal of Psychiatry 49, 751–752. 10.1177/0004867415584643 PMC4519522 25922354

[ref17] Kohn R, Saxena S, Levav I and Saraceno B (2004) The treatment gap in mental health care. Bulletin of the World Health Organization 82, 858–866. 15640922 PMC2623050

[ref18] Koike S, Yamaguchi S, Ojio Y, Shimada T, Watanabe KI and Ando S (2015) Long-term effect of a name change for schizophrenia on reducing stigma. Social Psychiatry and Psychiatric Epidemiology. 50, 1519–1526. 25947634 10.1007/s00127-015-1064-8

[ref19] Koike S, Yamaguchi S, Ojio Y, Ohta K, Shimada T, Watanabe K, Thornicroft G and Ando S (2018 *a*) A randomised controlled trial of repeated filmed social contact on reducing mental illness-related stigma in young adults. Epidemiology Psychiatric Sciences 27, 199–208. 27989255 10.1017/S2045796016001050 PMC7032789

[ref20] Koike S, Yamaguchi S, Ojio Y and Ando S (2018 *b*) Social distance toward people with schizophrenia is associated with favorable understanding and negative stereotype. Psychiatry Research 261, 264–268. 29329046 10.1016/j.psychres.2017.12.081

[ref21] Kutcher S, Wei Y and Morgan C (2016 *a*) Mental health literacy in post-secondary students. Health Education Journal 75, 689–697.

[ref22] Kutcher S, Wei Y and Coniglio C (2016 *b*) Mental health literacy: Past, Present, and Future. The Canadian Journal of Psychiatry 61, 154–158. 27254090 10.1177/0706743715616609 PMC4813415

[ref23] Lebowitz MS and Ahn WK (2012) Combining biomedical accounts of mental disorders with treatability information to reduce mental illness stigma. Psychiatric Services 63, 496–499. 22388477 10.1176/appi.ps.201100265 PMC3489926

[ref24] Lebowitz MS and Appelbaum PS (2018) Biomedical explanations of psychopathology and their implications for attitudes and beliefs about mental disorders. Annual Review of Clinical Psychology 15, 555–577. 10.1146/annurev-clinpsy-050718-095416 PMC6506347 30444641

[ref25] McLuckie A, Kutcher S, Wei Y and Weaver C (2014) Sustained improvements in students’ mental health literacy with use of a mental health curriculum in Canadian schools. BMC Psychiatry 14, 379. 25551789 10.1186/s12888-014-0379-4 PMC4300054

[ref26] Milin R, Kutcher S, Lewis SP, Walker S, Wei Y, Ferrill N and Armstrong MA (2016) Impact of a mental health curriculum on knowledge and stigma among high school students: a randomized controlled trial. Journal of the American Academy of Child and Adolescent Psychiatry 55, 383–391. e1. 27126852 10.1016/j.jaac.2016.02.018

[ref27] Rüsch N, Angermeyer MC and Corrigan PW (2005) Mental illness stigma: concepts, consequences, and initiatives to reduce stigma. European Psychiatry 20, 529–539. 16171984 10.1016/j.eurpsy.2005.04.004

[ref28] Rüsch N, Evans-Lacko SE, Henderson C, Flach C and Thornicroft G (2011) Knowledge and attitudes as predictors of intentions to seek help for and disclose a mental illness. Psychiatric Services 62, 675–678. 21632739 10.1176/ps.62.6.pss6206_0675

[ref29] Schnyder N, Panczak R, Groth N and Schultze-Lutter F (2017) Association between mental health-related stigma and active help-seeking: systematic review and meta-analysis. British Journal of Psychiatry 210, 261–268. 10.1192/bjp.bp.116.189464 28153928

[ref30] Schomerus G, Schwahn C, Holzinger A, Corrigan PW, Grabe HJ, Carta MG and Angermeyer MC (2012) Evolution of public attitudes about mental illness: a systematic review and meta-analysis. Acta Psychiatrica Scandinavica 125, 440–452. 22242976 10.1111/j.1600-0447.2012.01826.x

[ref32] Tanaka G (2003) Development of the mental illness and disorder understanding scale. International Journal of Japanese Sociology 12, 95–107.

[ref33] Thornicroft G, Rose D, Kassam A and Sartorius N (2007) Stigma: ignorance, prejudice or discrimination? British Journal of Psychiatry 190, 192–193. 10.1192/bjp.bp.106.025791 17329736

[ref34] Thornicroft G, Mehta N, Clement S, Evans-Lacko S, Doherty M, Rose D, Koschorke M, Shidhaye R, O'Reilly C and Henderson C (2016) Evidence for effective interventions to reduce mental-health-related stigma and discrimination. Lancet 387, 1123–1132. 26410341 10.1016/S0140-6736(15)00298-6

[ref35] Ungar T, Knaak S and Szeto AC (2016) Theoretical and practical considerations for combating mental illness stigma in health care. Community Mental Health Journal 52, 262–271. 26173403 10.1007/s10597-015-9910-4 PMC4805707

[ref36] Watson AC, Otey E, Westbrook AL, Gardner AL, Lamb TA, Corrigan PW and Fenton WS (2004) Changing middle schoolers’ attitudes about mental illness through education. Schizophrenia Bulletin 30, 563–572. 15631245 10.1093/oxfordjournals.schbul.a007100

[ref37] Wei Y, Hayden JA, Kutcher S, Zygmunt A and McGrath P (2013) The effectiveness of school mental health literacy programs to address knowledge, attitudes and help seeking among youth. Early Intervention in Psychiatry 7, 109–121. 23343220 10.1111/eip.12010

[ref38] Whiteford HA, Degenhardt L, Rehm J, Baxter AJ, Ferrari AJ, Erskine HE, Charlson FJ, Norman RE, Flaxman AD, Johns N, Burstein R, Murray CJ and Vos T (2013) Global burden of disease attributable to mental and substance use disorders: findings from the Global Burden of Disease study 2010. Lancet 382, 1575–1586. 23993280 10.1016/S0140-6736(13)61611-6

[ref39] Yamaguchi S, Mino Y and Uddin S (2011) Strategies and future attempts to reduce stigmatization and increase awareness of mental health problems among young people: a narrative review of educational interventions. Psychiatry and Clinical Neurosciences 65, 405–415. 21851449 10.1111/j.1440-1819.2011.02239.x

[ref40] Yamaguchi S, Wu SI, Biswas M, Yate M, Aoki Y, Barley EA and Thornicroft G (2013) Effects of short-term interventions to reduce mental health-related stigma in university or college students: a systematic review. Journal of Nervous and Mental Disease 201, 490–503. 10.1097/NMD.0b013e31829480df 23719324

[ref41] Yamaguchi S, Koike S, Watanabe K and Ando S (2014) Development of a Japanese version of the reported and intended behaviour scale: reliability and validity. Psychiatry and Clinical Neurosciences 68, 448–455. 24920378 10.1111/pcn.12151

[ref42] Yamaguchi S, Ojio Y, Ando S, Bernick P, Ohta K, Watanabe KI, Thornicroft G, Shiozawa T and Koike S (2019) Long-term effects of filmed social contact or internet-based self-study on mental health-related stigma: a 2-year follow-up of a randomised controlled trial. Social Psychiatry Psychiatric Epidemiology 54, 33–42. 30315333 10.1007/s00127-018-1609-8

