# Supplementary material for: Effects of biomedical messages and expert-recommended messages on reducing mental health-related stigma: a randomised controlled trial
Source: Epidemiol Psychiatr Sci. 2019 Nov 22;29:e74. doi: 10.1017/S2045796019000714 (PMC8061129; doi:10.1017/S2045796019000714)
Supplement: Supplementary file 1 [file epssup.zip › S2045796019000714sup003.docx]

| Table S1. Effects of BMM and RCM interventions on stigma | | | | | | | |
| --- | --- | --- | --- | --- | --- | --- | --- |
|  | Biomedical messages  video lecture (BMM) Mean ± SD  (Effect size)^a)^ | | Recommended messages video lecture (RCM)  Mean ± SD  (Effect size)^a)^ | | Main effect of Time | Main effect of Group^b)^ | Time x Group interaction^b)^ |
|  |  |  |  |  | B [95% CI]  p value | B [95% CI]  p value | B [95% CI]  p value |
| MIDUS |  |  |  |  |  |  |  |
| Baseline | 16.60 ± 6.93 | | 17.84 ± 7.53 | | 0 (ref) | 1.24 [–0.83, 3.31] p = .23 | 0 (ref) |
| Post-test | 10.49 ± 6.12  (0.93) | | 12.57 ± 7.27  (0.71) | | **–6.11 [–7.42, –4.81]**  **p < .001** |  | 0.84 [–1.01, 2.69] p = .37 |
| 1-month follow-up | 11.04 ± 7.07  (0.79) | | 14.17 ± 8.71  (0.45) | | **–5.87 [–7.44, –4.30]**  **p < .001** |  | 2.20 [–0.02, 4.42] p = .05 |
| 1-year follow-up | 14.34 ± 8.58  (0.29) | | 16.43 ± 8.28 (0.18) | | **–2.56 [–4.27, –0.85]**  **p < .01** |  | 0.67 [–1.78, 3.13] p = .59 |
| RIBS-J future |  | |  | |  |  |  |
| Baseline | 12.49 ± 3.73 | | 12.04 ± 3.61 | | 0 (ref) | –0.44 [–1.44, 0.55] p = .38 | 0 (ref) |
| Post-test | 14.30 ± 3.23  (0.52) | | 14.57 ± 3.71 (0.69) | | **1.81 [1.22, 2.40]**  **p < .001** |  | 0.72 [–0.11, 1.55]  p = .09 |
| 1-month follow-up | 14.07 ± 2.91 (0.47) | | 13.74 ± 3.23 (0.50) | | **1.51 [0.90, 2.12]**  **p < .001** |  | 0.24 –0.62, 1,11] p = .58 |
| 1-year follow-up | 13.10 ± 3.47 (0.17) | | 11.88 ± 4.02 (0.04) | | 0.68 [–0.06, 1.43]  p = .07 |  | –0.72 [–1.80, 0.35] p = .19 |
| RIBS-J past |  | |  | |  |  |  |
| Baseline | 0.62 ± 0.89 | | 0.79 ± 1.06 | | 0 (ref) | 0.16 [–0.12, 0.45] p = .26 | 0 (ref) |
| 1-year follow-up | 0.81 ± 1.09 (0.19) | | 0.57 ± 0.92 (0.22) | | 0.15 [–0.05, 0.34] p = .14 |  | **–0.38 [–0.66, –0.10]**  **p < .01** |
| B: Non-standardized regression coefficients; SD: Standard deviation; CI: Confidence interval; ref: Reference; RIBS-J: Japanese version of Reported and Intended Behaviour Scale; MIDUS, Mental Illness and Disorder Understanding Scale;  a) Cohen’s d, d = (*M*_Timet_ – *M_Time_*_0_) ⁄ *SD*_pooled,_ *SD*_pooled_ = √((*SD*_Timet_^2^ + *SD*_Time0_^2^) ⁄ 2), Timet = 1 (post-test survey), 2 (1-month follow-up survey), or 3 (1-year follow-up survey); b) Reference group is BMM group | | | | | | | |

| Table S2. Effects of BMM and RCM interventions on the MIDUS subscale | | | | | | | |
| --- | --- | --- | --- | --- | --- | --- | --- |
|  | Biomedical messages  video lecture (BMM) Mean ± SD  (Effect size)^a)^ | | Recommended messages video lecture (RCM)  Mean ± SD  (Effect size)^a)^ | | Main effect of Time | Main effect of Group^b)^ | Time x Group interaction^b)^ |
|  |  |  |  |  | B [95% CI]  p value | B [95% CI]  p value | B [95% CI]  p value |
| MIDUS_TR |  |  |  |  |  |  |  |
| Baseline | 3.67 ± 2.65 | | 4.03 ± 3.10 | | 0 (ref) | 0.37 [–0.47, 1.20] p = .39 | 0 (ref) |
| Post-test | 3.18 ± 2.81  (0.18) | | 2.11 ± 2.68  (0.66) | | –0.49 [–1.04, 0.06]  p = .08 |  | **-1.43 [–2.22, -0.65] p < .001** |
| 1-month follow-up | 3.12 ± 2.78  (0.20) | | 3.46 ± 3.75  (0.17) | | –0.66 [–1.35, 0.03]  p = .06 |  | 0.17 [–0.80, 1.15] p = .73 |
| 1-year follow-up | 3.84 ± 3.73  (0.05) | | 3.66 ± 3.22 (0.12) | | 0.10 [–0.64, 0.84]  p = .79 |  | -0.49 [–1.55, 0.57] p = .36 |
| MIDUS_EM |  | |  | |  |  |  |
| Baseline | 9.81 ± 4.15 | | 10.26 ± 3.95 | | 0 (ref) | 0.40 [–0.74, 1.54] p = .49 | 0 (ref) |
| Post-test | 5.26 ± 3.40  (1.21) | | 8.42 ± 4.27 (0.45) | | **-4.60 [-5.35, -3.86]**  **p < .001** |  | **2.76 [1.71, 3.81]**  **p < .001** |
| 1-month follow-up | 5.59 ± 3.54 (1.10) | | 7.89 ± 4.28 (0.58) | | **-4.37 [-5.17, -3.56]**  **p < .001** |  | **1.89 [0.75, 3.03] p < .01** |
| 1-year follow-up | 7.53 ± 3.71 (0.58) | | 9.43 ± 4.84 (0.19) | | **-2.53 [-3.48, -1.58]**  **p < .001** |  | **1.39 [0.03, 2.75] p < .05** |
| MIDUS_SR |  | |  | |  |  |  |
| Baseline | 3.09 ± 2.26 | | 3.55 ± 2.54 | | 0 (ref) | 0.46 [–2.00, 1.12] p = .17 | 0 (ref) |
| Post-test | 2.06 ± 1.87  (0.50) | | 2.07 ± 2.37 (0.60) | | **-1.03 [-1.49, -0.58]**  **p < .001** |  | -0.43 [-1.07, 0.22]  p = .19 |
| 1-month follow-up | 2.33 ± 2.09 (0.35) | | 2.82 ± 2.63 (0.28) | | **-0.84 [-1.36, -0.32]**  **p < .01** |  | 0.12 [-0.61, 0.85] p = .75 |
| 1-year follow-up | 2.97 ± 2.61 (0.05) | | 3.34 ± 2.35 (0.09) | | -0.15 [-0.70, 0.40]  p = .60 |  | -0.19 [-0.97, 0.60] p = .64 |
| B: Non-standardized regression coefficients; SD: Standard deviation; CI: Confidence interval; ref: Reference; MIDUS, Mental Illness and Disorder Understanding Scale; MIDUS_TR: Treatability of illness of MIDUS; MIDUS_EM: Efficacy of medication of MIDUS; MIDUS_SR: Social Recognition of MIDUS; a) Cohen’s d, d = (*M*_Timet_ – *M_Time_*_0_) ⁄ *SD*_pooled,_ *SD*_pooled_ = √((*SD*_Timet_^2^ + *SD*_Time0_^2^) ⁄ 2), Timet = 1 (post-test survey), 2 (1-month follow-up survey), or 3 (1-year follow-up survey); b) Reference group is BMM group | | | | | | | |

| Table S3. Changes of proportion of mental health-related experience in this trial | | | | |  |
| --- | --- | --- | --- | --- | --- |
|  | Biomedical messages video lecture (BMM) | | Recommended messages video lecture (RCM) | | *Statistical values*^a)^ |
|  | Baseline n = 90 | 1-year follow-up n = 73 | Baseline n = 89 | 1-year follow-up  n = 68 |  |
|  |  |  |  |  |  |
| Having mental health problems, n (%) | 21 (23.33) | 22 (30.14) | 24 (26.97) | 15 (22.06) | *χ^2^= 0.81, p = 0.34* |
| Receiving care/treatment from professionals, n (%) | 11 (12.22) | 13 (17.81) | 14 (15.73) | 12 (17.65) | *χ^2^= 0.00, p = 1.00* |
| Attending a lecture about mental health problems, n (%) | 38 (42.22) | 38 (52.05) | 31 (34.83) | 36 (52.94) | *χ^2^= 0.00, p = 1.00* |
| Viewing media describing an individual with mental health problems^b)^, n (%) | 72 (80.00) | 65 (89.04) | 65 (73.03) | 57 (83.82) | *χ^2^= 0.44, p = 0.46* |
| Seeking help from family members and close friends for mental health problems, n (%) | 24 (26.67) | 18 (24.66) | 21 (23.60) | 15 (22.06) | *χ^2^= 0.03, p = 0.84* |
| 1. Comparisons between groups at 1-year follow-up survey were tested using chi-square test. In addition, comparison of 1-year follow-up survey with baseline survey was tested using McNemar’s test. The statistical significance threshold was adjusted to 0.05/10 = 0.005 with Bonferroni correction. The proportions of "Attending a lecture about mental health problems" in RCM was significantly increased (χ^2^= 9.80, p = 0.003). 2. Media include television, newspapers, internet, etc. | | | | | |

| Table S4. Effects of BMM and RCM interventions on preference and intention to seek help | | | | | |
| --- | --- | --- | --- | --- | --- |
|  | Biomedical  messages  video lecture (BMM)  Mean ± SD  (Effect size)^a)^ | Recommended messages video lecture (RCM)  Mean ± SD  (Effect size)^a)^ | Main effect of Time^a)^ | Main effect of Group^b)^ | Time x Group interaction^b)^ |
|  |  |  | B [95% CI]  p value | B [95% CI]  p value | B [95% CI]  p value |
| Intention to seek-help |  |  |  |  |  |
| Baseline | 3.76 ± 0.93 | 3.73 ± 0.89 | 0 (ref) | –0.03 [–0.28, 0.23] p = .85 | 0 (ref) |
| Post-test | 3.98 ± 0.94 (0.23) | 4.09 ± 0.87 (0.41) | **0.22 [0.07, 0.37]**  **p < .01** |  | 0.14 [–0.07, 0.35] p = .20 |
| 1-month follow-up | 3.79 ± 0.90 (0.03) | 3.70 ± 0.94 (0.03) | 0.12 [–0.05, 0.29]  p = .17 |  | –0.15 [–0.39, 0.91] p = .22 |
| 1-year follow-up | 3.88 ± 0.92  (0.13) | 3.85 ± 1.03 (0.12) | 0.13 [–0.09, 0.36]  p = .25 |  | 0.04 [–0.29, 0.36] p = .83 |
| Intention to disclose |  |  |  |  |  |
| Baseline | 3.12 ± 1.55 | 2.97 ± 1.59 | 0 (ref) | –0.16 [–0.60, 0.28] p = .49 | 0 (ref) |
| Post-test | 3.31 ± 1.61 (0.12) | 3.47 ± 1.62 (0.31) | 0.19 [–0.02, 0.40]  p = .08 |  | **0.32 [0.02, 0.62] p < .05** |
| 1-month follow-up | 3.39 ± 1.49 (0.18) | 3.26 ± 1.63 (0.18) | **0.33 [0.04, 0.63]**  **p < .05** |  | –0.01 [–0.43, 0.41] p = .97 |
| 1-year follow-up | 3.14 ± 1.55  (0.01) | 3.16 ± 1.67 (0.12) | -0.01 [–0.40, 0.37]  p = .95 |  | 0.18 [–0.38, 0.75] p = .52 |
| B: Non-standardized regression coefficients; SD: Standard deviation; CI: confidence interval; ref: Reference;  a: Cohen’s d, d = (*M*_Timet_ – *M_Time_*_0_) ⁄ *SD*_pooled,_ *SD*_pooled_ = √((*SD*_Timet_^2^ + *SD*_Time0_^2^) ⁄ 2), Timet = 1 (post-test survey), 2 (1-month follow-up survey),  or 3 (1-year follow-up survey); b: Reference group is BMM group | | | | | |
