# Supplementary figures and images for: Effects of biomedical messages and expert-recommended messages on reducing mental health-related stigma: a randomised controlled trial
Source: Epidemiol Psychiatr Sci. 2019 Nov 22;29:e74. doi: 10.1017/S2045796019000714 (PMC8061129; doi:10.1017/S2045796019000714)

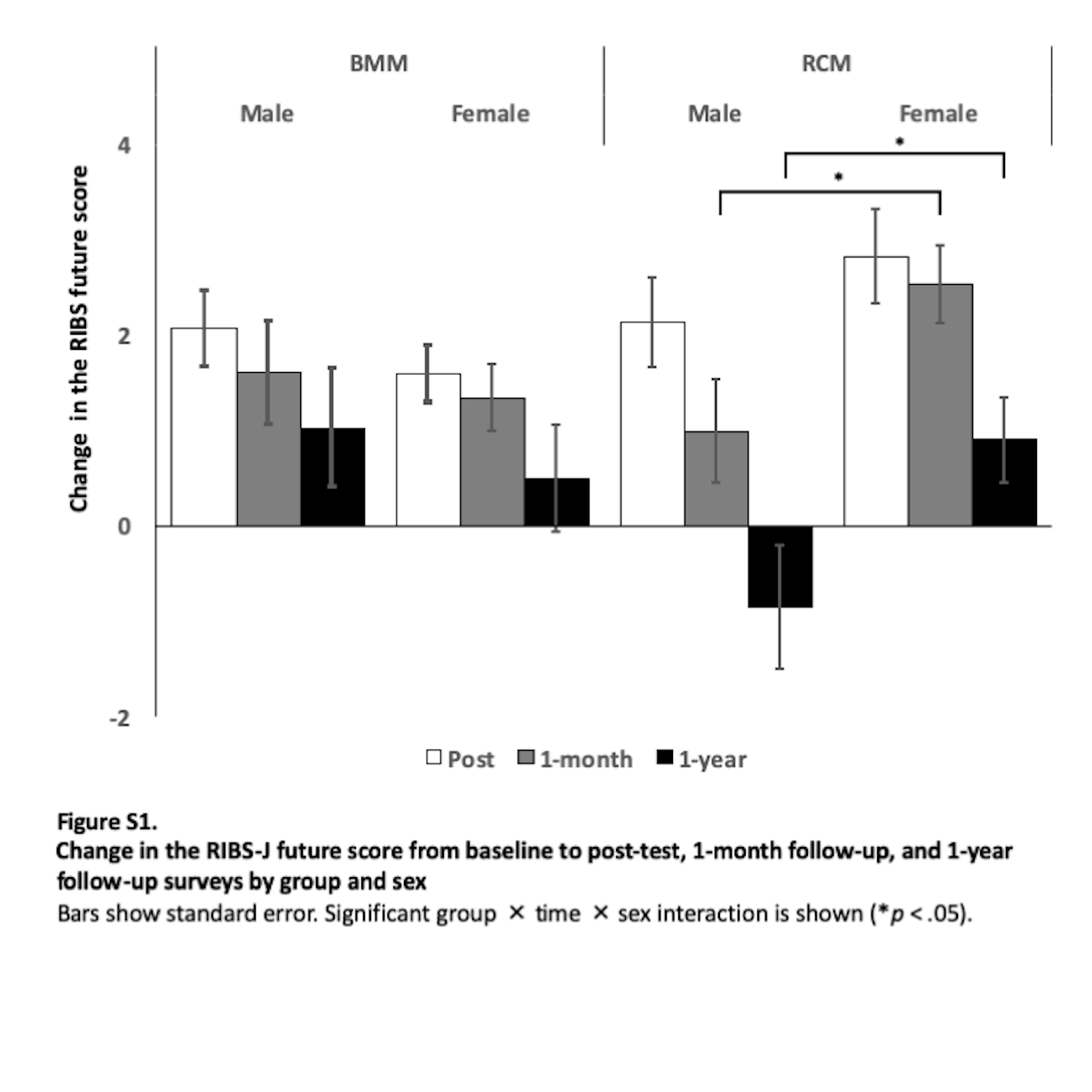

Supplement: Supplementary file 1 [file epssup.zip › S2045796019000714sup001.tiff]

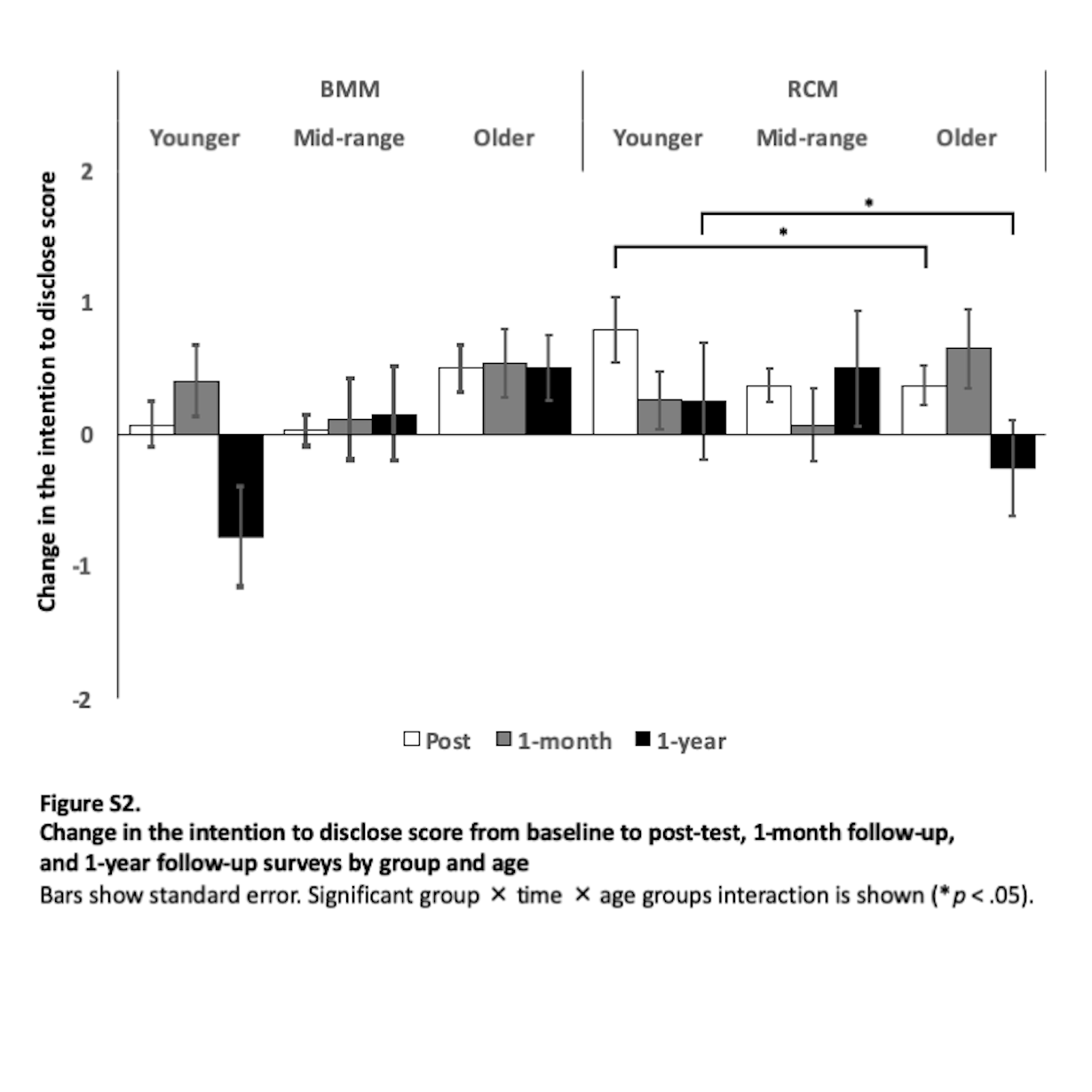

Supplement: Supplementary file 1 [file epssup.zip › S2045796019000714sup002.tiff]
